# A Foreign Body in the Cephalic Vein: Broken Piece of Intravenous Cannula

**DOI:** 10.7759/cureus.18813

**Published:** 2021-10-16

**Authors:** Ayesha Masood, Muhammad Jamil Malik, Muhammad Irfan Khan

**Affiliations:** 1 Vascular Surgery, Combined Military Hospital Rawalpindi, Rawalpindi, PAK

**Keywords:** retained cannula, catheterization, cephalic vein, fractured intravenous cannula, intravascular foreign body, peripheral/adverse effects"

## Abstract

Peripheral intravenous cannulation is a routine in the medical field with the rarity of complications in expert hands. However, at times, complications arise including the fracture of the cannula inside the vein, which is a rare but potentially serious complication with the possibility of pulmonary embolism. We have reported a case of a broken piece of a cannula in the cephalic vein removed with the help of a Fogarty catheter with the emphasis on preoperative imaging studies to localize it and use of a tourniquet to avoid distal migration during retrieval. There are varied reports about the conservative vs operative approaches for foreign bodies in vasculature. It should be removed in the first place where expertise allow so that the rare but potentially serious complications can be avoided.

## Introduction

Peripheral intravenous cannulation is a very common procedure performed in routine medical practice [1]. Catheters, cannulas, shrapnels, metallic fragments, and needles can embolize distally along the blood flow leading to lethal pulmonary embolisms in addition to infection, bleeding and thrombosis [2]. There are rare instances where broken pieces of intravenous cannulation are left inside the vein. It is of utmost importance to correctly locate the foreign body with imaging before attempting to remove it [3,4]. There are a variety of techniques to remove intravascular foreign bodies with minimally invasive techniques being first-line options nowadays [5].

## Case presentation

**Figure 1:**
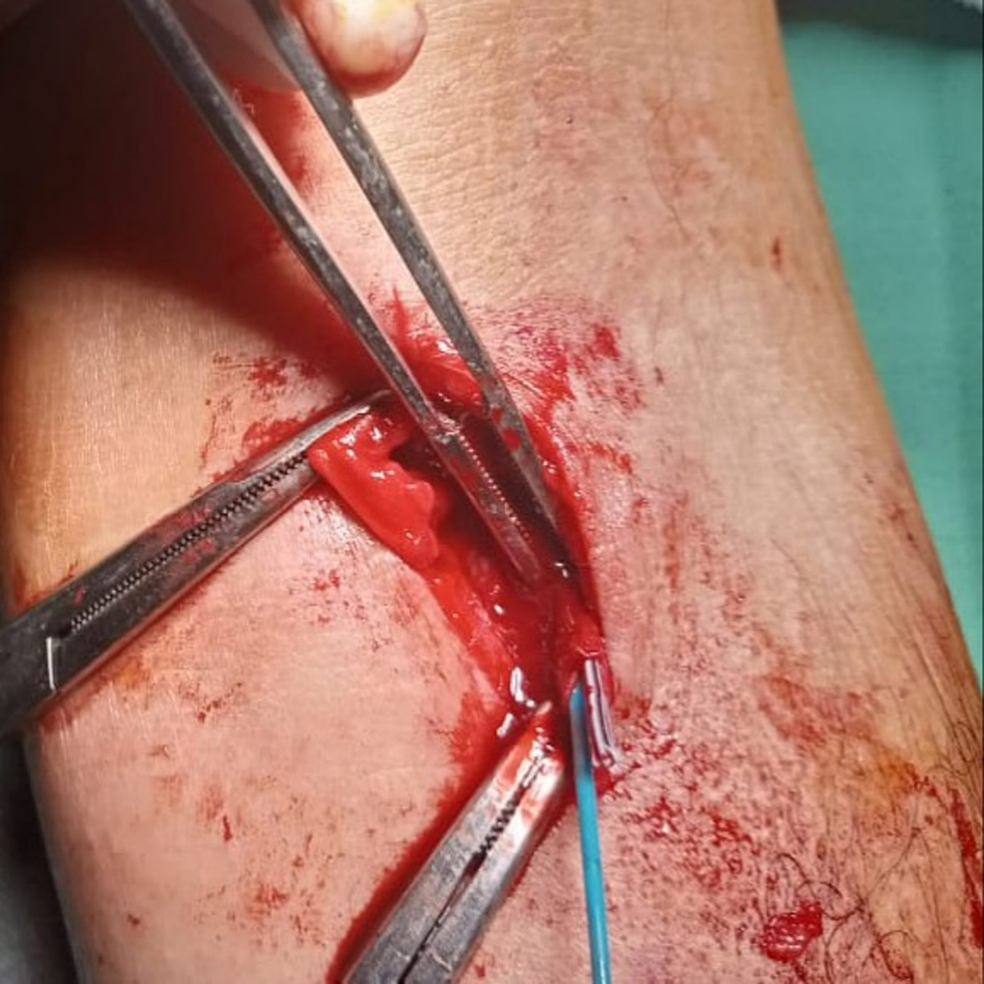
Venotomy with the retrieval of a cannula

**Figure 2:**
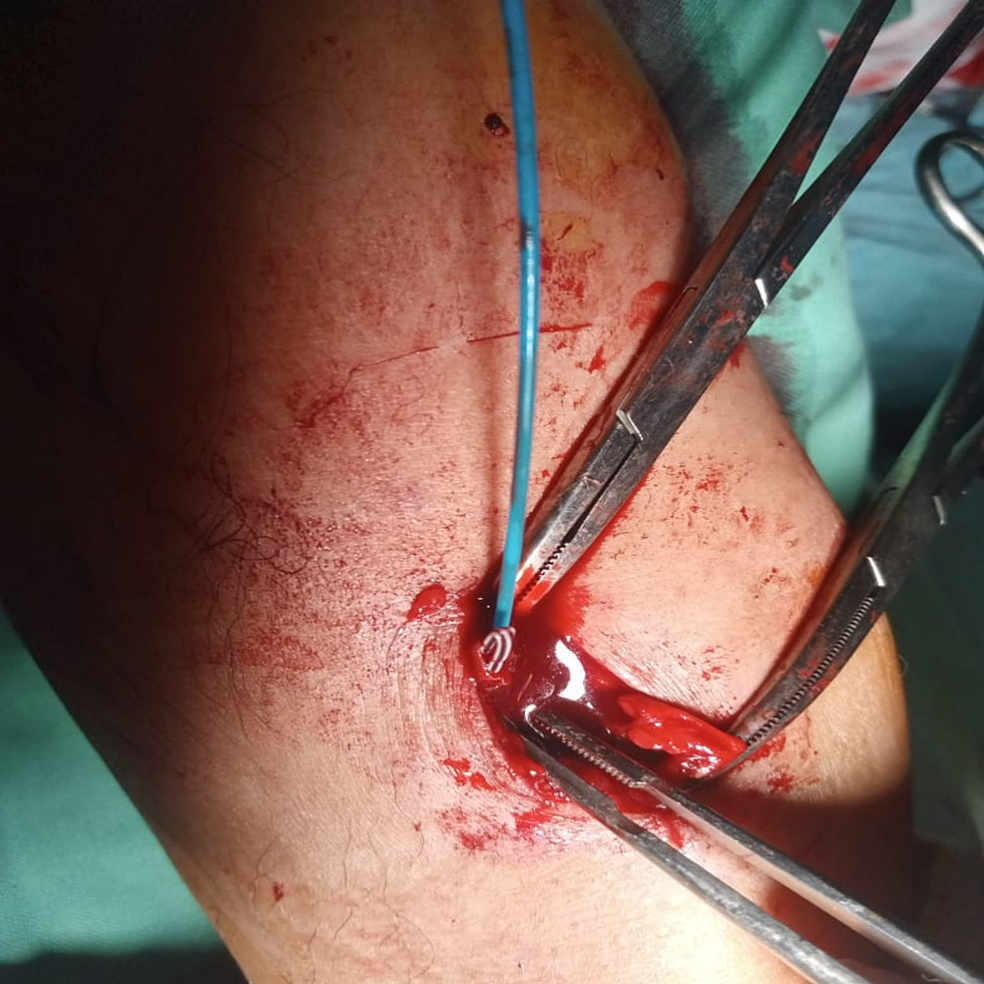
Retrieval with Fogarty catheter

We present a case of 40 years old male with a history of chest pain for which he was admitted and peripheral intravenous catheterization (PIVC) performed at the level of left cubital fossa to relieve pain. At the time of discharge, intravenous cannulation removal was attempted but it could not be retrieved completely. The patient developed pain and feeling of retained foreign body inside the peripheral vein of the cubital fossa. The patient was referred to the vascular surgery department for retrieval of a broken piece of an intravenous cannula. On examination, there was a mark of PIVC. However broken piece was not clearly palpable. X-ray study showed no clue as it was not radiopaque. Ultrasonography partially located the cannula piece inside the cephalic vein proximal to the cubital fossa. CT angiography was not available at that moment. The patient was shifted to operation theatre after informed consent for retrieval. A tourniquet was placed proximal to ultrasound marking. Cephalic vein was exposed, venotomy performed, the cannula was not clinically palpable so 4 Fr Fogarty catheter was used to retrieve the cannula from the distal site of cephalic vein almost 13cm from cubital fossa (Figures 1, 2).

**Figure 3:**
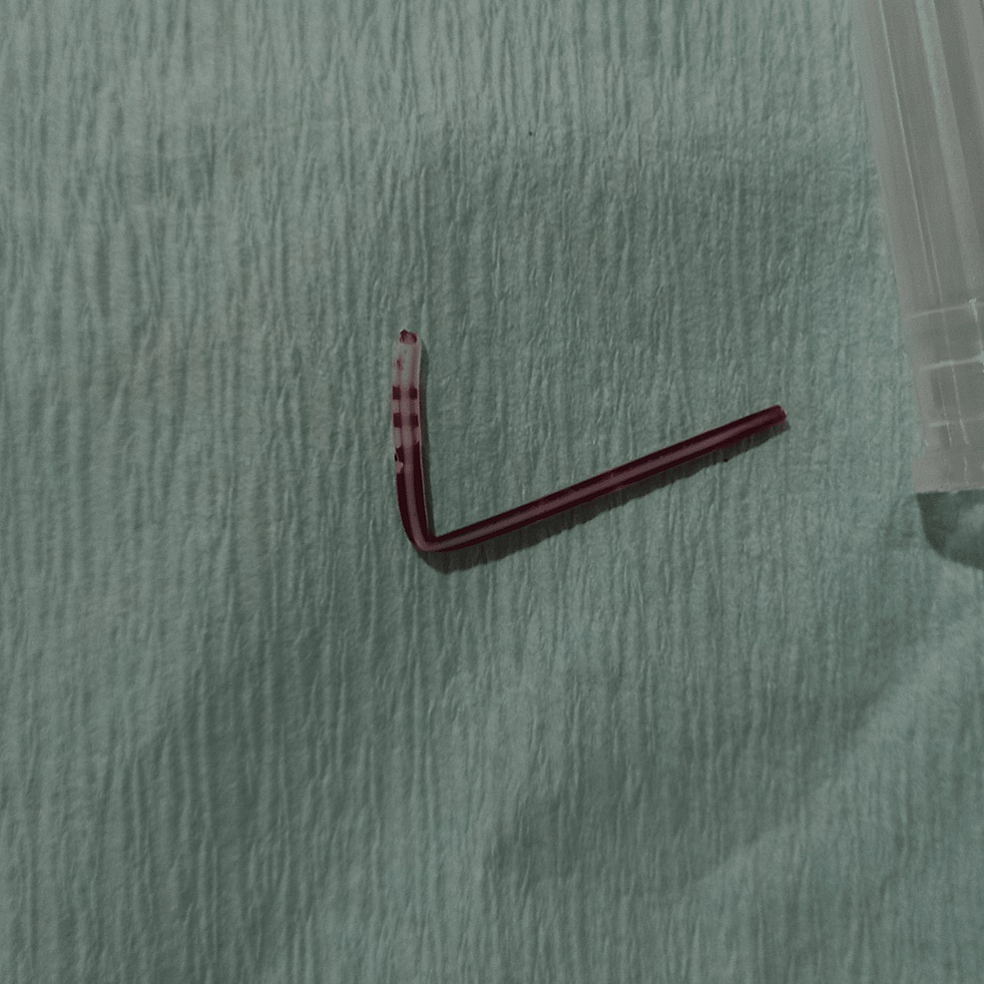
Broken piece of an intravenous cannula

Vein ends ligated, hemostasis secured and wound closed with uneventful follow-up after one week. Investigation of the cause of broken fragments (Figure 3) revealed that it could be either partial transaction due to reinsertion of the needle into already advanced cannula resulting in bending of the cannula and on the removal of cannula complete transaction took place.

It is important to avoid reinsertion of the needle into a partially advanced cannula.

## Discussion

Peripheral intravenous cannulation is a routine procedure with a rare possibility of fracture and retention of broken pieces inside the vein. There are various reports related to fracture of the central venous catheter and peripheral cannulation fracture with possible thrombosis, sepsis, migration, and air embolism [6,7]. The first reported case was spontaneous fracture and migration of central venous cannulation by Turner et al. in 1954 [1,6]. In our case, we used a 4Fr Fogarty catheter to retrieve the broken piece. There are various percutaneous, endovascular techniques namely loop snare, angioplasty balloon catheters, dormia basket, and retrieval forceps techniques [5]. The decision to extract foreign bodies is individualized depending upon various studies, with regular follow-up with imaging techniques if conservative management is opted [7]. It can go unrecognized for a significant time period with a mortality rate of 1.8% [8].

## Conclusions

Peripheral intravascular cannulation should be done with care and following the guidelines. Diagnosis of broken cannula inside the peripheral vein needs careful history and examination for a possible mechanism of the fractured cannula. Before retrieval, imaging investigations for localization and use of tourniquets are crucial to avoid distal migration with grave outcomes. Given the variable but potentially lethal outcomes of intravascular migration, early exploration should be the first choice.
